# Diseases of the Lachrymal Sac
*A Paper read before the Bristol Medico-Chirugical Society on Wednesday, February 13th, 1935.


**Published:** 1935

**Authors:** A. E. Iles

**Affiliations:** Ophthalmic Surgeon, Bristol General Hospital; Surgeon, Bristol Eye Hospital


### DISEASES OF THE LACHRYMAL SAC.*

BY

A. E. Iles, O.B.E., M.B., F.R.C.S.,

*Ophthalmic Surgeon, Bristol General Hospital ;*
*Surgeon, Bristol Eye Hospital.*

---

In dealing with the treatment of these conditions I propose to devote my time chiefly to the methods of treatment of stenosis and inflammation of the lachrymal passages, passing quickly over those of congenital or traumatic origin.

1. *Congenital.* — The most frequent cause is obstruction at the lower end owing to a mass of epithelial cells situate at the nasal opening. This results in the formation in most cases of a mucocele. Treatment consists either (1) of Crigler's method, which by means of pressure occlusion of the canaliculi forces the contents of the sac down the canal and breaks down the obstruction, or if this does not suffice, then (2) the passage of a probe under an anæsthetic will ensure a cure, especially if the probe be rotated so as to completely open the lower end of the canal.

2. *Traumatic.*—(*a*) Those due to wounds of the

* A Paper read before the Bristol Medico-Chirugical Society on Wednesday, February 13th, 1935.

lid. When the canaliculus has been torn across this calls for very careful approximation of the wound with a probe passed into the punctum through both segments of the canaliculus. (*b*) Laceration of the sac due to severe contusions of the face, where the firmly adherent fascia which overlies the sac is torn. These accidents give rise to those symptoms of surgical emphysema where the eye bulges forwards on the nose being blown, due to air being forced up the lachrymal passages from the nose. Treatment consists of pressure over the sac.

### Stenosis of Parts of the Lachrymal Passages.

Before dealing with stenosis and inflammation of the sac it may be just as well to go through the sequence of events which takes place in these cases.

First of all there is a slight obstruction which is intermittent, giving rise to occasional watering. As this obstruction becomes more definite the epiphora becomes more constant. Eventually stasis occurs, with the result that owing to irritation by decomposition the sac wall becomes inflamed, so that a chronic dacryocystitis with enlargement and loss of elasticity of the sac wall supervenes. Then if one presses over the sac regurgitation in the early stages of glairy fluid and latterly mucopus through the punctum takes place. As the lower end of the canal is continuous with the nasal mucosa, diseases of the nasal mucous membrane are very prone to infect the canal. In some cases this leads on to an acute dacryocystitis owing to infection of the surrounding tissues caused by a slight break in the sac lining.

(*a*) *The Canaliculus.*—In these cases it is advisable to dilate the passages rather than have recourse to canaliculus slitting, which is deleterious in case an operation such as Toti is required, as it has been found that these operations have a much more successful result if the valve-like action of the punctum is intact.

How does stricture occur, and is it frequent? In my experience there are many more cases in women than men, partly owing to the fact that the nasal canal in woman is narrower. It is more common than is supposed, and this is supported by Sandermann, who examined canaliculi and lachrymal canals in the cadaver. He found 80 per cent. had normal canaliculi, but in only 31 per cent. were the lachrymal passages really normal, forty cases being male and three female.

The first symptom is usually intermittent watering of the eyes in cold winds, frequently due to a slight obstruction. If this obstruction be allowed to persist stasis occurs, and then the stricture gets tighter and tighter. In early cases of obstruction my practice has always been, first of all, to pass a syringe into the canaliculus, and then endeavour to pass fluid into the nose. This should pass freely, and one can roughly measure the amount of obstruction by the force necessary for fluid to pass. Elschnig introduced a syringe in 1926 by which one was able to measure the amount of pressure required. Syringing in early cases may be sufficient by itself to bring about a cure. If fluid does not pass I then, after cocainization, endeavour to pass a probe through the canal. In these cases gentleness in manipulation is required, and if one allows the probe to rest with gentle pressure against the stricture frequently after a short interval the probe will be felt to pass on. This is aided by oil and adrenalin.

Lipoidol in localizing the stricture is also useful, as it should in a normal canal pass through in eight minutes, and X-ray will show the site of the stricture and also will show whether there are any diverticula present which would contraindicate the use of a probe.

X-ray examination of the region is helpful in that it may show up an aberrant cell. I had one of my own cases which illustrated this. On a West operation being performed, the rhinologist, Mr. Wright, found that he was in an accessory ethmoidal cell, and this had to be traversed before the sac was found.

Passage of an electric current to facilitate probing has been tried and stated to be of service, but I have had no experience of this. Forcible probing has been carried out by the use of large probes, and success has been claimed for this; but I feel that the large amount of bruising and, occasionally, even fracture of the bone which may result makes a trial of this method inadvisable.

I have frequently found that if probing is started sufficiently early in the course of the trouble it will result in a cure. In some cases even after a long history repeated probing will materially benefit the patient and allay the epiphora.

(*b*) *Slitting of the Canaliculus* used to be practised most extensively, but in my opinion it should be reserved for those cases when the puncta are displaced by ectropion, and thus a lake is made for the tears before they can get to the punctum.

### CHRONIC DACRYOCYSTITIS.

There are two methods of dealing with this :—

1. *Conservative.*—If a chronic dacryocystitis be present then first of all it is essential to try and clean the sac. Methods which have been applied are injections of silver nitrate in $\frac{1}{4}$ per cent. solution. This is only advisable when free entry without force can be made into the sac, one of which consists of injecting the fluid through a hollow probe.

2. *Operative.*—In my own practice, if there is a chronic dacryocystitis without much secretion and there is a mucocele formed so that without undue force one is unable to express the fluid up or down, I generally aspirate the sac directly through the skin by a hypodermic needle, and then inject into the sac a small quantity of 1 per cent. mercurochrome.

A solution of tincture of iodine has also been used in this way with beneficial results. I myself have had quite a number of successful cases, the most striking being a pregnant woman of eight months, who had a very large mucocele. I aspirated and left mercurochrome in the sac, with the result that when I saw her some months after there was no sign of obstruction and no palpable sac.

I have had an equal number of failures; but in view of the successes I am convinced that this is worth trying, in that you do no harm, you may cure, and you do not diminish the prospect of a cure by operative measures if necessary later. This method is useful if you are dealing with a severe ulcer of the cornea, in that it cleans up the sac temporarily.

Those cases of chronic dacryocystitis which do not respond to conservative treatment divide themselves into three groups :—

1. Those with obstructed canaliculi.

2. (*a*) Those with free canaliculi and with no matting around due to a previous dacryocystitis; (*b*) as in (*a*) but with considerable matting.

In the first group excision of the sac will probably give the best results, as with dacryocystorhinostomy a good passage from the puncta is required. If possible I always prefer restoration of the original channel, or provision of an equally efficacious alternative one, rather than excision of the sac, which should be confined to those matted sacs where it is very difficult to identify the walls.

*Excision of the sac* is very greatly favoured by many surgeons, and it entails a deeper dissection and disturbance of parts than the modified Toti operation, which does not necessitate the cutting across of the internal palpebral ligament, which is necessary in excision in order to get to the dome of the sac.

Since I am of the opinion that this disease in the majority of cases has primarily a nasal origin—some observers make it 78 per cent.—I make it my practice to have these cases seen by a rhinologist to report on the nasal condition.

By means of dacryocystorhinostomy a new opening is made into the nose, which in the majority of instances has proved successful. The disappearance of stasis is a great factor in clearing up the condition, and the conjunctival sac rapidly becomes sterile.

### ACUTE DACRYOCYSTITIS.

The acute form only supervenes on the chronic. The patient usually comes with a tense red swelling over the site of the sac, induration having extended beyond the sac owing to a loss of continuity in its walls.

As a rule these conditions are best treated by incision and cleaning up of the sac cavity, but there is so much matting present that excision of the diseased sac becomes advisable later on. Therefore early recognition and treatment of a chronic dacryocystitis should prevent this occurrence.

### SUMMARY.

Early recognition of lachrymal obstruction and treatment by gentle probing or syringing should bring about a cure.

When definite obstruction is present with a chronic dacryocystitis then restoration of the original channel or formation of an alternative one should be procured.

With considerable matting of the surrounding tissues and impermeable canaliculi extirpation of the sac should be performed. It has been said that a good extirpation of the sac is better than a poor dacryocystorhinostomy; but the converse is equally true, and the constant epiphora of one of my friends brings this home to me every time I see him.

I feel I cannot do better than conclude by quoting

Professor Meller, himself an exponent of excision, who ends by saying, "The aim of the treatment of chronic dacryocystitis must be the same ideal which we try to attain in the treatment of all diseases —restoration of the normal anatomic and physiological conditions, that is, in this particular case restoration of the mucus membrane of the sac and duct to a normal condition, and restoration of the normal avenues of conveyance so that they become permeable again."

